# Late neointimal volume reduction is observed following biodegradable polymer-based drug eluting stent in porcine model

**DOI:** 10.1016/j.ijcha.2021.100792

**Published:** 2021-05-11

**Authors:** Takeshi Ijichi, Gaku Nakazawa, Sho Torii, Hirofumi Nagamatsu, Ayako Yoshikawa, Shintaro Nakamura, Junko Souba, Atsushi Isobe, Hitomi Hagiwara, Yuji Ikari

**Affiliations:** a Department of Cardiology, Tokai University, School of Medicine, Kanagawa, Japan; b Department of Cardiology, Kindai University, Faculty of Medicine, Osaka, Japan; c TERUMO Corporation Evaluation Center, Kanagawa, Japan

**Keywords:** Biodegradable polymer-based drug-eluting stents, Durable polymer-coated everolimus-eluting stents, Optical frequent domain imaging

## Abstract

**Background:**

The BP-SES has an abluminally applied biodegradable polymer that is fully resorbed after 3–4 months but may have longer-lasting effects. The aim of this study was to determine the long-term vascular response to the novel Ultimaster™ sirolimus-eluting stent (BP-SES).

**Methods:**

BP-SESs, everolimus-eluting stents (DP-EESs), and bare metal stents were implanted in 22 coronary arteries of 15 mini-swine. All animals underwent optical frequent domain imaging (OFDI) to assess neointimal volume and quality at either 1 (n = 7) or 3 (n = 8) months and at 9 (n = 15) months and were euthanized at 9 months. Stents were subsequently histologically investigated to analyze the vascular response and maturity of neointimal tissue according to cell density.

**Results:**

OFDI revealed greater regression in neointimal volume from 3 to 9 months with BP-SESs than with DP-EESs (−0.6 ± 0.5 mm^2^ vs. 0.00 ± 0.4 mm^2^, *p* = 0.07). Although there was no significant difference between BP-SESs and DP-EESs in the inflammation score (BMS, BP-SES, and DP-EES: 0.1 ± 0.1, 0.3 ± 0.4, and 0.4 ± 0.4, respectively; *p* < 0.0001) in histological analysis, BP-SESs showed slightly greater maturity than DP-EESs (1.8 ± 0.3, 1.7 ± 0.3, and 1.6 ± 0.3, *p* = 0.09).

**Conclusions:**

While both BP-SESs and DP-EESs showed minimal inflammatory responses at 9 months, BP-SESs showed a trend for greater neointimal maturity and regression, which may be related to earlier completion of the vascular response.

## 1 Introduction

In the percutaneous coronary intervention field, drug-eluting stents (DESs) have improved clinical outcomes by suppressing neointimal growth after stent implantation. However, several new concerns have emerged due to delayed arterial healing and stent endothelialization after DES implantation, including stent thrombosis, vessel remodeling, and restenotic late catch-up [1], [2], [3], [4], [5], [6], [7], [8], [9], [10].

Chronic complications after DES implantation include the late catch-up phenomenon, linked to excessive neointimal proliferation in the stented segment, and late stent thrombosis [3], [4], [11]. Long-term follow-up of second-generation durable polymer-coated everolimus-eluting stents (DP-EESs) revealed significantly reduced stent thrombosis, target vessel revascularization, and myocardial infarction versus other stents [12]. While DP-EESs show lower stent thrombosis rates than first-generation DESs in the clinical setting, the prevalence of restenosis and arterial injury of DP-EESs is histologically comparable to that of first-generation DESs [13]. Some important issues remain, such as increased inflammation, particularly with durable polymers. These adverse complications can be somewhat attributed to the duration of hypersensitivity reactions and the inflammatory response after stenting, and stent polymers may be the stent type most strongly linked to these late adverse events [3], [11], [14]. Indeed, after complete elution of the anti-proliferative drug, the persistence of a permanent polymer can induce local hypersensitivity and inflammatory reactions [15].

Recently, novel biodegradable polymers (BPs) have been proposed, as well as low-profile, cobalt, or platinum chromium stent backbones; these stents are likely associated with an improved vascular response [16], [17]. In contrast to DP-EESs, the polymers of BP-DESs eventually dissolve, leaving behind a bare metal stent (BMS) [18], [19]. Previous pooled analyses found that the risks of target lesion revascularization (hazard ratio 0.82, 95% confidence interval 0.68 to 0.98, *p* = 0.029) and very late stent thrombosis (hazard ratio 0.22, 95% confidence interval 0.08 to 0.61, *p* = 0.004) were significantly lower in patients with a BP-DES than in those with a durable polymer DES [20], [21], [22], [23]. Therefore, BPs may partly help to reduce the late catch-up phenomenon.

A clinical trial reported that comparable vessel healing was achieved after abluminal BP-coated sirolimus-eluting stent (BP-SES) implantation [24]. The CENTURY II trial□(BP-SES vs. DP-EES) demonstrated comparable clinical outcomes (target lesion revascularization and very late stent thrombosis) with both stents up to five years of follow-up [25]. However, full elucidation is required of the detailed long-term tissue vascular response after BP-DES implantation. We thus evaluated the long-term vascular healing after BMS, BP-SES, and DP-EES implantation in a porcine coronary artery model.

## 2 Materials and methods

### 2.1 Study design

**Fig. 1:**
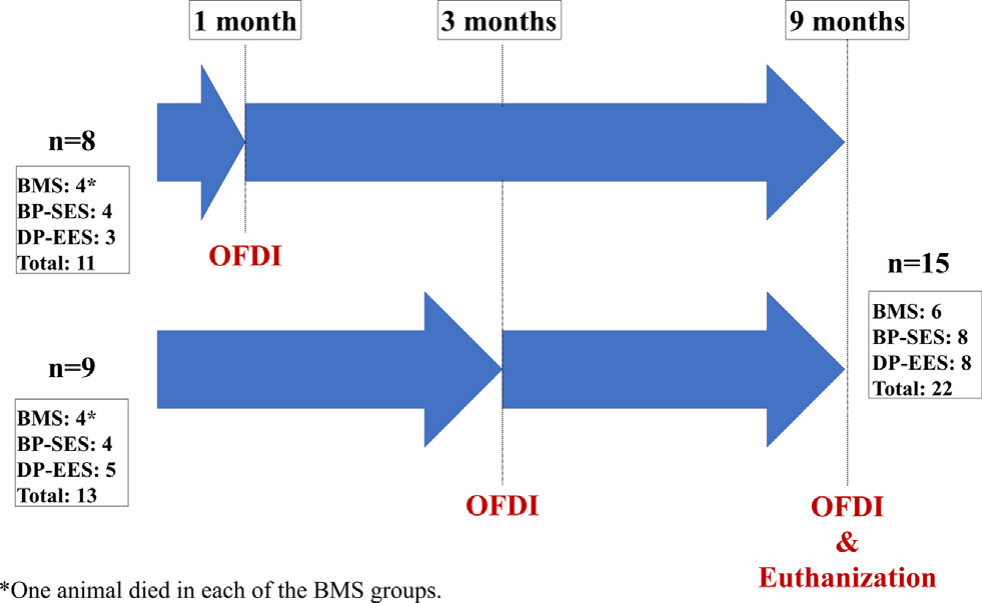
**Study Flow Chart.** All animals underwent OFDI at either 1 or 3 months and at 9 months. After euthanasia at 9 months, stents were histologically investigated to analyze the vascular response and its maturity. One animal died before the euthanasia in each of the 2 BMS groups.

Three different types of DESs were implanted into 17 mini-pigs (Japan Farm Co., Ltd., Kagoshima, Japan) (Fig. 1): DP-EESs (XIENCE Prime™; Abbott Vascular, Tokyo, Japan; n = 8), BP-SESs (Ultimaster™; Terumo Corp., Tokyo, Japan; n = 8), and BMSs (Kaname; Terumo Corp.; n = 8), with 1 of each type implanted into each pig (1 stent per vessel). The DP-EES is an everolimus-eluting stent with a uniform coating of durable fluoropolymer (polyvinylidene fluoride-co-hexafluoropropene) [26]. The BP-SES has an abluminal coating with a matrix containing sirolimus and poly (DL-lactide)-co-caprolactone [27], [28]. The BP is completely metabolized into carbon dioxide and water within 3–4 months. The number of animals required was determined from previous work [29]. All animals were sacrificed at 9 months to investigate the early vascular response. The stents were then histologically examined. This study was approved and performed according to the guidelines of the Institutional Animal Care and Use Committee of R&D Headquarters at Terumo Corp.

### 2.2 Animal preparation and procedures

The current study was based on US Food and Drug Administration guidance. Briefly, the animals’ clinical conditions were observed at least once per day, except on non-workdays, during the period from the day before the implantation to the scheduled day of euthanization. The mini-pigs were visually observed and observations recorded, including external appearance, appetite, respiratory condition, nutrition status, and fecal condition, as well as any other abnormalities. All animals were individually housed in pens for pigs. About 1.5 kg of forage for miniature swine (M-16; CLEA Japan, Inc.) was provided once daily.

All animals were administered oral clopidogrel (75 mg/day) and aspirin (330 mg/day) from 3 days before the procedure until the day before euthanization. The animals were also fasted for more than 15 h from the day before the procedure. After 2%–4% sevoflurane anesthesia, surgical access was obtained via the carotid artery using general sterile techniques. During cardiac catheterization, heparin (300 IU/kg) was given to maintain an adequate activated clotting time. Vessel allocation to experimental groups was predetermined to distribute the different stent types equally among 3 different coronary arteries, with a targeted oversize of 1.1–1.2:1 [29]. After stent implantation, coronary angiography was performed to ensure vessel patency and the absence of coronary arterial dissection.

The animals were euthanized under general anesthesia at 9 months. Their hearts were excised and pressure-perfused with 0.9% saline until clear of blood, followed by pressure perfusion fixation in 10% neutral buffered formalin until hardening of the heart muscle was evident.

### 2.3 OFDI imaging acquisitions and assessments

All stent segments were imaged with optical frequent domain imaging (OFDI) to assess lumen and stent area, stenotic area, neointimal volume, and stent strut coverage at either 1 (n = 10) or 3 (n = 12) months and at 9 months (Fig. 1). A 0.014-inch guide wire was introduced into the vessel followed by an OFDI catheter (FastView; Terumo Corp.) and sequential images were acquired at a pullback rate of 20 mm/s (8 frames/mm). Image analysis was conducted as in a previous study [30]. For quantitative analysis, cross-sectional OFDI images were analyzed at 1-mm intervals. For image matching, we used the distance from the stent edge and landmarks such as side branches to match the location of the cross-sections among the 1-, 3-, and 9-month examinations. Struts were classified as uncovered if any part of the strut was visibly exposed to the lumen and as covered if a tissue layer was visible over all reflecting surfaces. Neointimal thickness was measured from the stent strut to the vessel–lumen border (neointimal surface or strut surface if uncovered) for each stent strut. The frequency of uncovered struts (%Uncovered struts) was calculated as the number of uncovered struts divided by the total number of struts for each stent. To assess the unevenness of neointimal thickness, a neointimal unevenness score was calculated for each cross-section as the maximum neointimal thickness in 1 cross-section divided by the average neointimal thickness of the same cross-section.

**Fig. 2:**
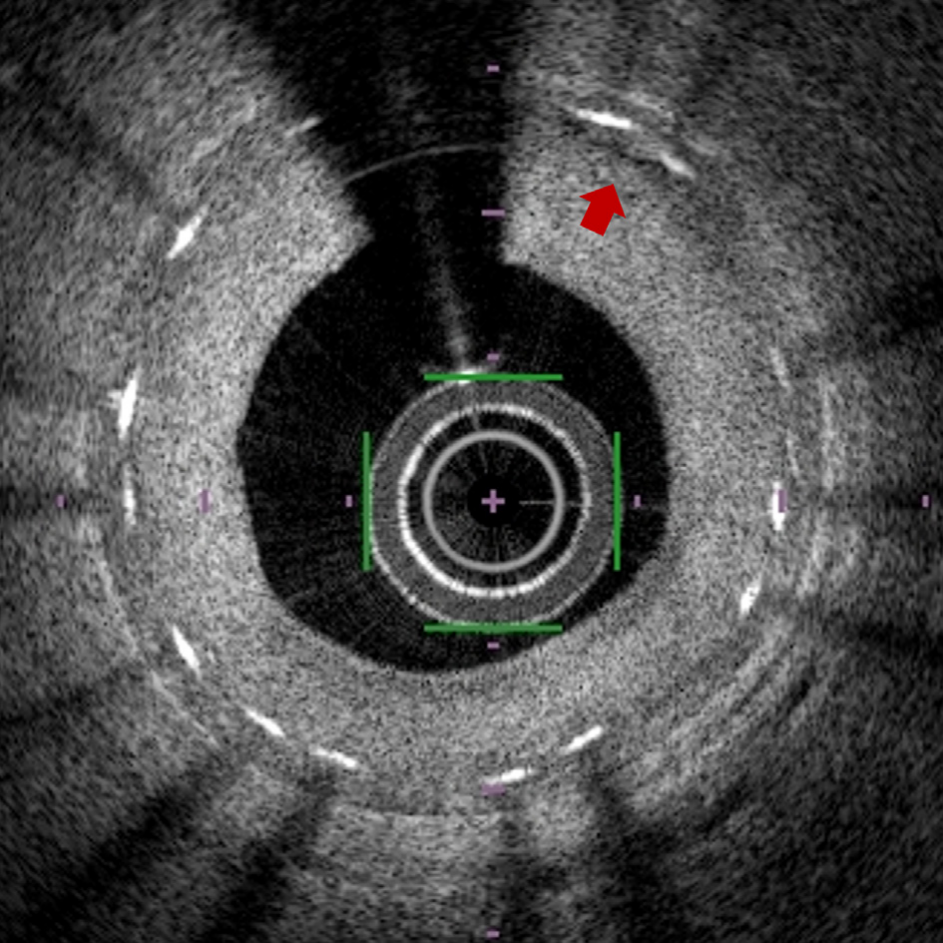
**Representative Image of the Peri-strut Low-intensity Area.** A representative image of the peri-strut low-intensity area (red arrow). (For interpretation of the references to colour in this figure legend, the reader is referred to the web version of this article.)

The peri-strut low-intensity area (PLIA) was also analyzed for each stent strut (Fig. 2). The PLIA, which is a potential marker of delayed arterial healing, was defined as the region around stent struts with a homogeneous lower-intensity appearance than the surrounding tissue without significant signal attenuation behind the area [31]. PLIA was measured as previously described [31]. Briefly, the percentage of stent struts with a PLIA (%PLIA) was calculated as the number of struts with a PLIA divided by the number of visible struts × 100.

### 2.4 Histologic preparation and assessments

Stented arteries were fixed in 10% neutral buffered formalin and embedded in Quetol-651 resin. After polymerization, the arteries were divided into proximal, middle, and distal blocks, serially sectioned, and then stained with hematoxylin and eosin and a combined Verhoeff and Masson trichrome stain [32].

Injury and inflammation scores were awarded as previously reported [33], [34]. Briefly, the injury score was determined as follows: 0 = no injury; 1 = break in the internal elastic membrane; 2 = perforation of the media; and 3 = perforation of the external elastic membrane to the adventitia. The inflammation score was graded as follows: 0 = no inflammatory cells surrounding the strut; 1 = light, noncircumferential lymphohistiocytic infiltrate surrounding the strut; 2 = localized, moderate-to-dense cellular aggregate noncircumferentially surrounding the strut; and 3 = circumferential dense lymphohistiocytic cell infiltration of the strut.

**Fig. 3:**
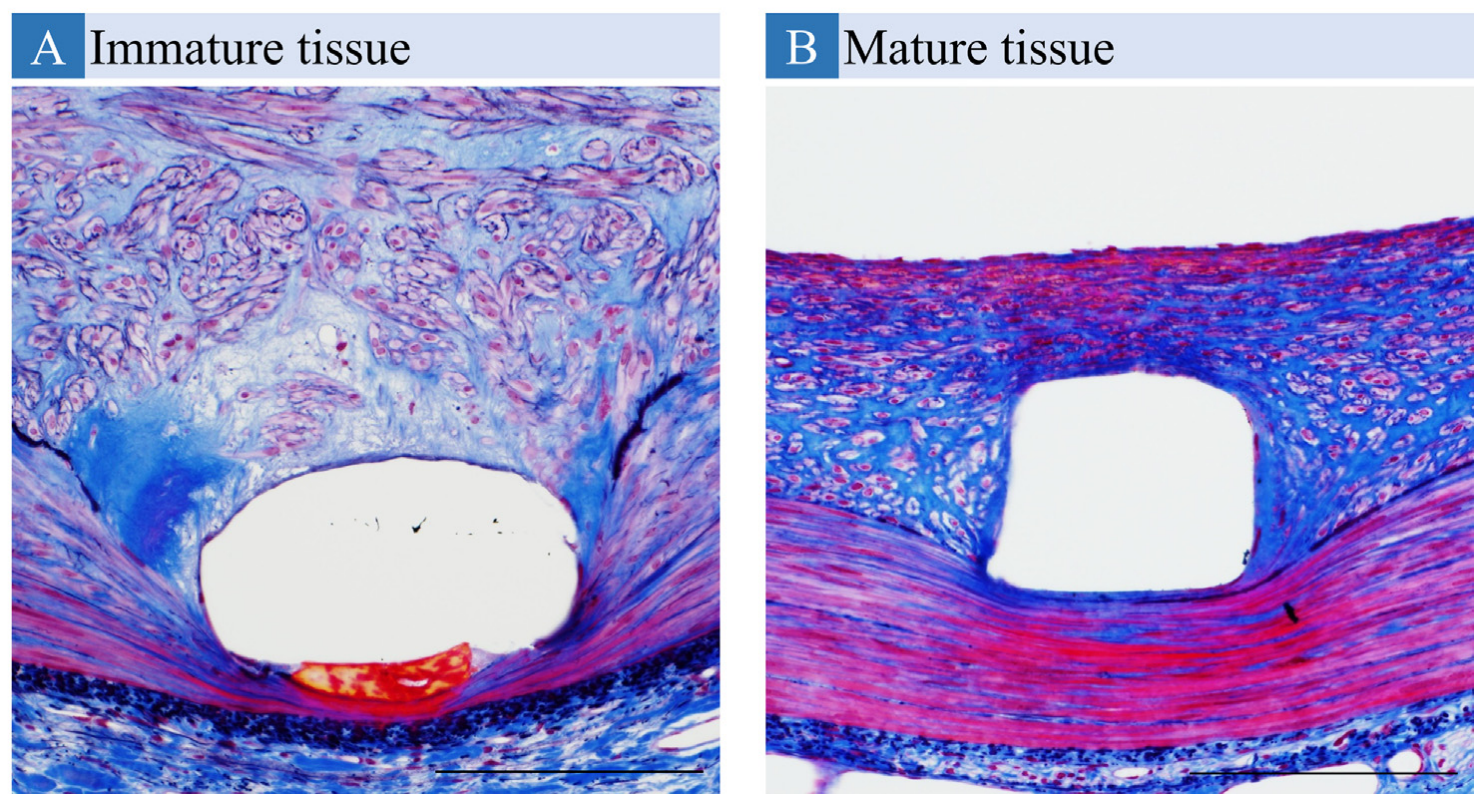
**Representative Histologic Sections Stained with a Combined Verhoeff and Masson Trichrome for Calculation of the Maturity Score.** Sections show a high-power (20×) magnification following combined Verhoeff and Masson trichrome staining. Immature tissue (score = 1) was defined as fiber-rich extracellular matrix with few smooth muscle cells. Inflammatory cells, macrophages, and fibrin were mainly observed surrounding stent struts (A). Mature tissue (score = 2) showed abundant smooth muscle cells in neointimal tissue with few inflammatory cells (B).

Mature neointimal tissue around the stent strut was defined as tissue with a predominance of smooth muscle cells and infrequent inflammatory cells, macrophages, fibrin, and proteoglycan [35], [36]. To assess the maturity of neointimal tissue, the maturity score was graded as: 1 (poor smooth muscle cell) = inflammatory cells, macrophages, and fibrin scattered around stent struts with infrequent smooth muscle cells; and 2 (rich smooth muscle cell) = neointimal tissue with rich smooth muscle cells with few inflammatory cells (Fig. 3).

In addition to these mean scores, the percent distribution in these scores was analyzed in individual struts from each stent to determine the variability in the vascular response among animals. The cross-sectional areas (internal elastic lamina and lumen) of each stented section were digitally detected and measured using digital morphometry (cellSens Standard; Olympus, Tokyo, Japan).

### 2.5 Statistics

JMP for Windows version 9.0.2 (SAS Institute Inc., Cary, NC, USA) and EXSUS version 7.7.1 (CAC EXICARE Corporation, Tokyo, Japan) were used for the analysis of histopathological findings. Data are expressed as mean ± standard deviation for continuous variables and as percentage and interquartile range for categorical variables. The distribution normality of continuous variables was assessed using Bartlett’s test for equal variances. Statistical comparisons were performed using ANOVA with Dunnett’s post hoc correction when data sets were normally distributed; otherwise, Kruskal-Wallis tests with a Steel test were used. Nonparametric score data, including vascular responses, %Uncovered struts, and %PLIA, were compared using a Mann-Whitney *U* test or Fisher’s exact test. A p value < 0.05 was considered statistically significant.

## 3 Results

### 3.1 Stent implantation

Stents were successfully implanted into 3 major coronary arteries of 17 pigs without any differences in quantitative coronary analyses. Two animals died unexpectedly following the anesthesia, 1 in each of the 2 BMS groups, leaving a total of 22 implants (in 15 pigs) available for follow-up at 9 months (Fig. 1). After necropsy, no abnormalities were found in the heart and other organs.

### 3.2 OFDI findings

**Fig. 4:**
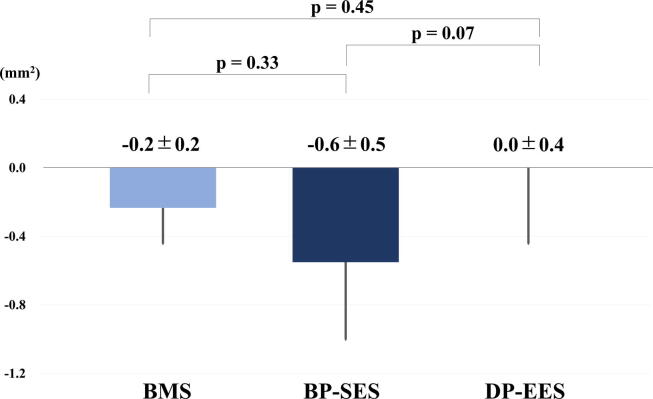
**Regression in the Neointimal Volume from 3 to 9 Months.** Regression in the neointimal volume was analyzed by OFDI at 3 and 9 months.

The OFDI findings are detailed in Table 1. While the average neointimal area was nonsignificantly larger in the BMS group than in the DES groups at 1 month (BMS, BP-SES, and DP-EES: 1.4 ± 0.5 mm^2^, 0.8 ± 0.3 mm^2^, and 1.0 ± 0.1 mm^2^, respectively), the BMS group had a lower average neointimal area than the DES groups at 3 months (1.6 ± 0.6 mm^2^, 2.1 ± 0.3 mm^2^, and 2.5 ± 0.4 mm^2^, respectively). Although the DP-EES group had a significantly higher neointimal area versus the BMS group at 9 months, no differences were detected between the BP-SES and DP-EES groups (1.4 ± 0.6 mm^2^, 1.6 ± 0.6 mm^2^, and 2.5 ± 0.8 mm^2^, respectively; *p* < 0.05). OFDI revealed nonsignificantly higher regression in neointimal volume from 3 to 9 months in the BP-SES group compared with the DP-EES group (−0.2 ± 0.2 mm^2^, −0.6 ± 0.5 mm^2^, and 0.0 ± 0.4 mm^2^; *p* = 0.07) (Fig. 4). The percentage of uncovered struts and the PLIA were nonsignificantly greater with DESs than with BMSs (Table 1). Similarly, among DESs, the %PLIA of struts was nonsignificantly higher with DP-EESs than with BP-SESs at each time point (Table 1).

**Table 1:** Comparison of OFDI findings.

Time point	1 month				3 months				9 months			
Stent type (number)	BMS	BP-SES	DP-EES	*p* value	BMS	BP-SES	DP-EES	*p* value	BMS	BP-SES	DP-EES	*p* value
	(n = 3)	(n = 4)	(n = 3)		(n = 3)	(n = 4)	(n = 5)		(n = 6)	(n = 8)	(n = 8)	
Lumen area (mm^2^)	2.9 ± 1.9	3.7 ± 0.6	4.7 ± 1.5	*p* = 0.33	2.7 ± 1.0	2.9 ± 1.1	2.4 ± 0.9	*p* = 0.79	3.3 ± 1.3	3.10 ± 1.2	3.1 ± 1.5	*p* = 0.93
Stent area (mm^2^)	4.2 ± 1.2	4.5 ± 0.4	5.7 ± 1.5	*p* = 0.25	4.3 ± 0.7	5.0 ± 0.8	4.9 ± 1.0	*p* = 0.58	4.4 ± 1.0	4.7 ± 0.8	5.2 ± 1.2	*p* = 0.28
Area of stenosis (%)	37.5 ± 23.3	18.6 ± 7.7	18.4 ± 4.6	*p* = 0.20	38.3 ± 15.5	44.3 ± 15.0	51.5 ± 10.0	*p* = 0.41	24.7 ± 15.7	35.2 ± 17.7	43.3 ± 17.0	*p* = 0.15
Average neointimal area (mm^2^)	1.4 ± 0.5	0.8 ± 0.3	1.0 ± 0.1	*p* = 0.15	1.6 ± 0.6	2.1 ± 0.3	2.5 ± 0.4	*p* = 0.08	1.4 ± 0.6	1.6 ± 0.7	2.5 ± 0.8	*p* < 0.05
% Uncovered struts (%)	0 (0–0)	0 (0–0.6)	0 (0–1.1)	*p* = 0.56	0 (0–0)	0 (0–0)	0 (0–0.5)	*p* = 0.50	0 (0–0)	0 (0–0)	0 (0–0)	*p* = 1.00
% PLIA (%)	0 (0–0.8)	0 (0–1.5)	0 (0–3.7)	*p* = 0.91	0 (0–0.7)	3.4 (0.8–7.6)	3.6 (1.0–12.4)	*p* = 0.20	0 (0–0)	0 (0–7.3)	1.0 (0–4.0)	*p* = 0.09
Values are presented as mean ± SD, the median (interquartile range) or number (percent); BMS, bare metal stent; BP-SES, abluminal biodegradable polymer-coated sirolimus-eluting stent; DP-EES, durable polymer-coated everolimus-eluting stent; PLIA, *peri*-strut low-intensity area.

### 3.3 Histomorphometric assessment and measurements

**Fig. 5:**
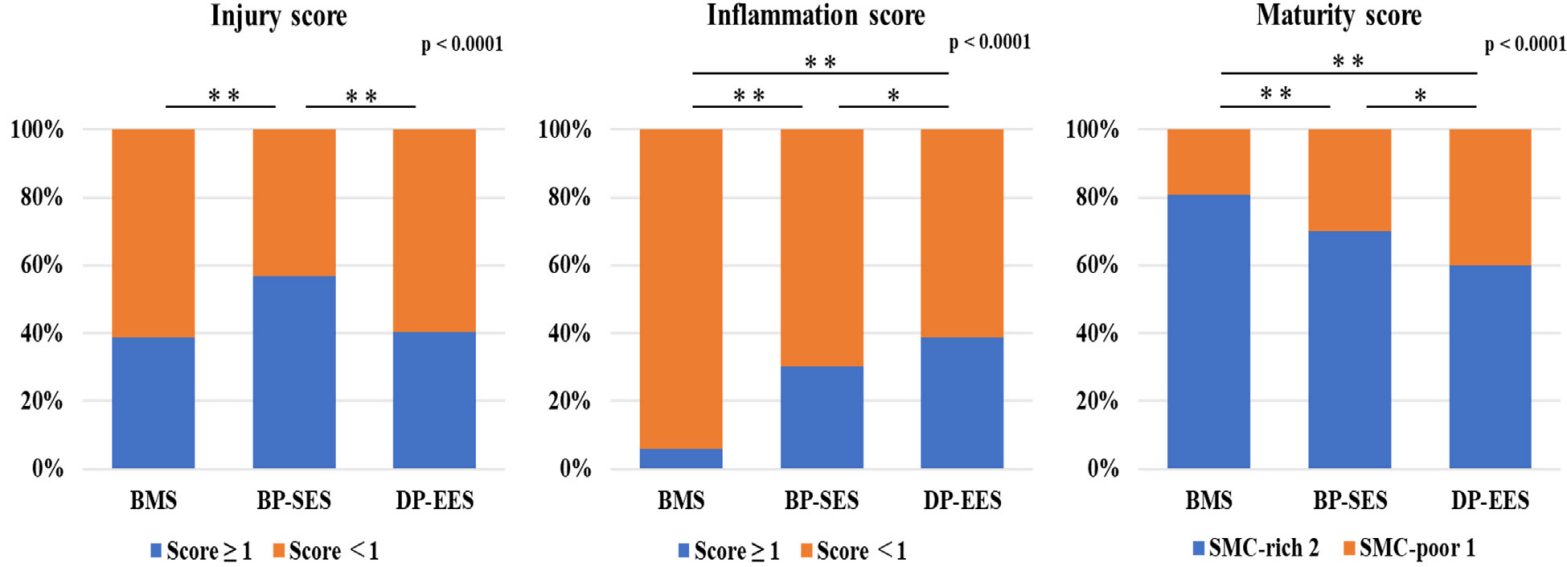
**Percent Distribution of the Vascular Response in Each Stent.** Histological distribution of the vascular response at 9 months. The vascular response was assessed using injury, inflammatory, and maturity scores. *p < 0.05, **p < 0.01.

The results of histomorphometric analyses are shown in Table 2. The injury score was slightly but nonsignificantly higher with BP-SESs than DP-EESs. The inflammation score was very low in all stent groups (BMS, BP-SES, and DP-EES: 0.1 ± 0.1, 0.3 ± 0.4, and 0.4 ± 0.4, respectively; *p* < 0.0001). Regarding the vascular response distribution, while the number of stent struts with an injury score greater than 1 was more frequently found with BP-SESs compared with the other stents (BMS, BP-SES, and DP-EES: injury score ≥ 1, 39%, 57%, and 40%, *p* < 0.0001) (Fig. 5), the numbers of stent struts with inflammation and maturity scores greater than 1 were higher in the DP-EES group (inflammation score ≥ 1, 6%, 30%, and 39%, *p* < 0.0001; maturity score 2, 81%, 70%, and 60%, *p* < 0.0001).

**Table 2:** Histological findings.

Stent type (number of struts)		BMS (288)	BP-SES (384)	DP-EES (360)	*p* value
Injury score	Mean	0.5 ± 0.6	0.8 ± 0.6	0.6 ± 0.6	*p* = 0.45
	0	176	166	215	
	1	69	147	75	
	2	40	68	70	
	3	3	3	0	

Inflammation score	Mean	0.1 ± 0.1	0.3 ± 0.4	0.4 ± 0.4	*p* < 0.01
	0	271	268	221	
	1	17	106	132	
	2	0	5	7	
	3	0	5	0	

Maturity score	Mean	1.8 ± 0.3	1.7 ± 0.3	1.6 ± 0.3	*p* = 0.09
	SMC-rich	233	269	216	
	SMC-poor	55	115	144	

Values are presented as mean ± SD or number (percent); BMS, bare metal stent; BP-SES, abluminal biodegradable polymer-coated sirolimus-eluting stent; DP-EES, durable polymer-coated everolimus-eluting stent; SMC, smooth muscle cell.

## 4 Discussion

The major findings of the current animal study are that BP-SESs, compared with DP-EESs, show (1) greater neointimal regression on OFDI from 3 months to 9 months; and (2) greater neointimal maturity.

While vessel wall impairment is correlated with restenosis in the BMS era, its impact has been minimized by the use of DESs, which is likely related to the use of powerful anti-proliferative drugs with prolonged release kinetics that profoundly inhibit the reparative response to arterial damage [5]. However, at the same time, hypersensitivity reactions related to drug toxicity and the polymer lead to vascular damage and inflammation after DES implantation. The degree of vascular damage has been reported to be highly dependent on the type of drug and its dose and release kinetics [37]. Durable polymers induce granulomatous and hypersensitivity reactions in animal models and these complications have also been observed with the use of first-generation SESs in humans at long-term follow-up [4], [5], [38]. Otsuka et al. [13] published an autopsy study comparing second-generation and first-generation DESs. A total of 204 lesions (SESs, 73; paclitaxel-eluting stents, 85; DP-EESs, 46) from 149 autopsy cases with a duration of implantation >30 days and ≤3 years were pathologically analyzed. The uncovered strut frequency was markedly lower with DP-EESs (2.6%) than with SESs (18.0%; *p* < 0.0005) and paclitaxel-eluting stents (18.7%; *p* < 0.0005). Regarding inflammation, DP-EESs showed significantly lower inflammation scores compared with SESs. However, Mori et al. [39] suggested that BP-SESs exhibited significantly faster endothelium coverage and higher expression of endothelial junctional VE-cadherin vs. DP-EESs.

Delayed healing, hypersensitivity reactions, and non-functional endothelium, or its insufficient restoration, along with specific patient/lesion characteristics, can somewhat be linked to polymer properties. BPs show superior antirestenotic efficacy to durable and polymer-free stents, without the long-term negative effects of persistent coverings [21]. In terms of the biological response, BP-SESs might have positive effects that suppress the injury and inflammation induced by polymer absorption within 3–4 months, after which time the DESs is expected to act more like BMSs [27]. At 9 months, the inflammatory reaction distribution was greater in the present study with DP-EESs than with BP-SESs. Importantly, the neointimal tissue maturation around the stent strut was the lowest in DP-EES vs. the other luminal polymer and non-coated stents. Consistent with these results, there was a trend toward a higher percentage of neointimal regression with BP-SESs on OFDI from 3 months to 9 months compared with DP-EESs. Interestingly, few in vivo results have been obtained on neointimal regression with BP-DESs. With respect to the EGO-Combo study (not compared with other durable polymer DESs), the Combo stent, an abluminal sirolimus-eluting coating composed of a BP, patients demonstrated neointimal regression from 9 to 24 months [40]. It is reasonable to assume that the potential benefits related to the use of a BP-DES might theoretically appear at a later time point.

The evidence thus far suggests that the neointima peaks 6–12 months after BMS implantation, with the neointimal volume subsequently decreasing as type III collagen is replaced by type I collagen [41]. These observations are consistent with clinical studies showing that the neointimal growth peaks 6 to 9 months after BMS implantation and then decreases slowly up to 3 years [42]. Meanwhile, histopathological analyses of human specimens have demonstrated that neointimal tissue after DES implantation can consist of heterogeneous components, including proteoglycan-rich tissue, organized thrombus, smooth muscle cells, atheroma, inflammatory cells, and fibrinoids [38]. Therefore, it could be hypothesized that these tissue types have different optical properties, resulting in a differential appearance on follow-up optical coherence tomography. In a previous preclinical study using a porcine stent implantation model [31], PLIA on optical coherence tomography images was demonstrated to represent fibrinogen deposition surrounded by hypocellular regions consisting of fibrin or proteoglycans, which are often observed in the healing process. The incidence of PLIA might be related to late target lesion revascularization [4], [5], [6], [38], [43], [44]. In the present study, while no significant difference was observed, there was a trend toward greater presence of PLIA on OFDI in DP-EESs vs. BP-SESs (Table 1). As a result, it could be assumed that the faster vascular healing contributed to the decreased presence of PLIA in the BP-SES group. Moreover, a previous study described that the incidence of DP-EES struts with PLIA decreased from the mid-phase (6–12 months) to late-phase (24 months) follow-up after stenting (6.2% ± 5.9% to 4.6% ± 4.5%, *p* = 0.01) [45], suggesting that further progressive vessel healing continued up to 2 years after stenting in the clinical setting.

These findings indicate a tendency for a better outcome with BP due to reduced late inflammatory reactions. However, it is still unclear whether the use of BP-coated DESs instead of other durable polymer-coated DESs will result in improved late outcomes in the clinical setting. Therefore, further long-term follow-up is needed to compare the long-term safety and efficacy of BP-SESs with those of other DESs. Ongoing studies have been designed to confirm the efficacy of BP-SESs among patients with acute myocardial infarction (MASTER study at 12 months, BP-SES vs. BMS: target vessel failure, 6.1% vs. 14.4%, p < 0.001) [46] and to investigate their performance in consecutive patients undergoing percutaneous coronary intervention in daily clinical practice (e-ULTIMASTER, NCT02188355).

There are several limitations to the present study. First, the favorable vascular compatibility of BP compared with permanent polymer in healthy porcine coronary arteries cannot be extrapolated to diseased human coronary arteries, where disease conditions and atherosclerotic plaque composition might influence polymer degradation and the inflammatory response [47]. Second, we examined a particular coating methodology and load of poly DL-lactide-co-caprolactone and durable fluoropolymer coatings, respectively, and these results might not be generalizable to other polymer coating methodologies and load doses. Third, the study was performed with a limited number of samples. It is likely that the lack of significance is related to the limited sample number, which should be addressed in a future histopathological study with sequential follow-up.

## 5 Conclusion

Our study demonstrated that abluminal polymer-coated SESs show greater neointimal maturity and regression, suggesting earlier completion of the vascular response.

## Declaration of Competing Interest

Gaku Nakazawa is a consultant for Japan Stent Technology, Boston Scientific, and Terumo Corporation, and is in receipt of research grants from Boston Scientific, Abbott Vascular, and Terumo Corporation; Junko Souba, Atsushi Isobe and Hitomi Hagiwara are employees of Terumo Corporation; Yuji Ikari is in receipt of research grants from Boston Scientific, Abbott Vascular, Kaneka Medics, Asahi Intech, Terumo Corporation; All other authors have no competing interests.
